# Phase-sensitive plasmonic biosensor using a portable and large field-of-view interferometric microarray imager

**DOI:** 10.1038/lsa.2017.152

**Published:** 2018-02-23

**Authors:** Filiz Yesilkoy, Roland A Terborg, Josselin Pello, Alexander A Belushkin, Yasaman Jahani, Valerio Pruneri, Hatice Altug

**Affiliations:** 1 Institute of BioEngineering, École Polytechnique Fédérale de Lausanne, CH-1015 Lausanne, Switzerland; 2 ICFO—Institut de Ciències Fotòniques, The Barcelona Institute of Science and Technology, 08860 Castelldefels (Barcelona), Spain; 3 ICREA—Institució Catalana de Recerca i Estudis Avançats, 08010 Barcelona, Spain

**Keywords:** interferometric imaging, label-free plasmonic biosensors, lens-free imaging, phase interrogation, point-of-care devices, protein microarray detection

## Abstract

Nanophotonics, and more specifically plasmonics, provides a rich toolbox for biomolecular sensing, since the engineered metasurfaces can enhance light–matter interactions to unprecedented levels. So far, biosensing associated with high-quality factor plasmonic resonances has almost exclusively relied on detection of spectral shifts and their associated intensity changes. However, the phase response of the plasmonic resonances have rarely been exploited, mainly because this requires a more sophisticated optical arrangement. Here we present a new phase-sensitive platform for high-throughput and label-free biosensing enhanced by plasmonics. It employs specifically designed Au nanohole arrays and a large field-of-view interferometric lens-free imaging reader operating in a collinear optical path configuration. This unique combination allows the detection of atomically thin (angstrom-level) topographical features over large areas, enabling simultaneous reading of thousands of microarray elements. As the plasmonic chips are fabricated using scalable techniques and the imaging reader is built with low-cost off-the-shelf consumer electronic and optical components, the proposed platform is ideal for point-of-care ultrasensitive biomarker detection from small sample volumes. Our research opens new horizons for on-site disease diagnostics and remote health monitoring.

## Introduction

Biosensing is one of the most sought-after applications in the field of nanophotonics because the enhanced light–matter interactions via sub-wavelength confinement and amplification of optical near-fields give access to unprecedented real-time, label-free and highly sensitive optical signal transductions^1, 2, 3, 4, 5, 6, 7, 8, 9, 10^. For example, nanoengineered plasmonic devices have revolutionized the performance of traditional Raman^11, 12^, fluorescent^13^ and infrared^14^ spectroscopy techniques enabling extraction of essential data on complex molecular structures, as well as single-molecule detection^15^. In particular, refractometric plasmonic sensors, such as those based on surface plasmon resonance (SPR)^16^ and localized-SPR (LSPR)^17, 18^ effects, have opened up new horizons in label-free optical biosensing, overcoming limitations of cumbersome and time-consuming labeled approaches, such as enzyme-linked immunosorbent assay and fluorescence detection. Moreover, the small-volume, near-field resonant plasmonic modes act as perfect probes to monitor minute local refractive index changes caused by molecular-binding events on the sensor surface. Most recently, facilitated by convenient far-field spectral intensity-measuring techniques, versatile on-chip plasmonic biosensors enabling clinically relevant detection limits of various biomaterials, such as bacteria^19^, viruses^20^, exosomes^21^ and proteins^22^, have been reported. Despite the recent progress, several challenges still exist for nanoplasmonic sensor technologies, including the need for simplified and robust optical readout systems and mass-scalable nanostructured chips with low manufacturing costs.

From a more applied perspective, on-site biosensing without dedicated personnel and infrastructure is essential for the biomedical community^23, 24, 25^. In particular, simple, robust and affordable bio-detection technologies that can rapidly and quantitatively detect multiple analytes within small sample volumes would not only benefit medical diagnostics but also other fields, such as food safety and environmental surveillance^26^. Several attempts have been made to engineer point-of-care (POC) biosensors that leverage nanoplasmonics^27, 28, 29, 30^. Most of these devices rely on amplitude (intensity) interrogation in which a narrow-band illumination source, such as a light-emitting diode (LED), is tuned to the plasmonic resonance, and the intensity variations due to the spectral resonance shifts that are induced by local refractive index changes are quantified on an imaging sensor, for example, a cellphone camera^31^, among other techniques^32, 33, 34^. Although this mode of detection can achieve spectral monitoring without the need for expensive and bulky spectrophotometers, its detection capabilities are limited by the plasmonic resonance mode properties, such as sensitivity to refractive index and quality factor (Q-factor, inversely proportional to the bandwidth of the mode) as well as the level of environmental and instrumental noise. Moreover, they do not usually allow multiplexed and high-throughput detection as the operational sensing area is limited either by the optical beam, when microscopes are used for read-out, or the size of the nanoplasmonic sensor, which is often small when fabricated using time-consuming and sophisticated techniques.

An alternative optical signal transduction mechanism for sensing is phase interrogation, which measures phase retardations of electromagnetic wavefronts. Using phase rather than intensity in optical detection has improved microscopy of thin and transparent objects^35, 36^ and elucidated the crystal structures of atomically thin layers in the field of X-ray diffraction^37^, among many other applications^38^. Phase-based detection was introduced into SPR technology by Kabashin *et al*^39^ to exploit phase shifts varying sharply at plasmonic resonances, first reported by Abeles *et al*^40^ Conventional light sources suffer from high-intensity (amplitude) noise, which in turn reduces the detection sensitivity of intensity-based plasmonic sensors. Instead, phase detection allows for lower noise and versatile signal-processing possibilities, such as spatial and temporal mapping due to the inherent relative measurement of phase with respect to a reference beam^41^. Moreover, in the traditional intensity-based setting, sensing is done at wavelengths that correspond to the side slopes of resonant modes rather than at center wavelengths where the intensity varies minimally as the resonance shifts. Despite that, the side slopes of the intensity spectrum change moderately when compared to the abrupt change that the phase spectrum exhibits at the center of the modes. Therefore, the phase response allows for superior refractive index sensing^42^.

Within the context of phase interrogation, nanostructured plasmonic surfaces are still mostly unexplored and their phase response investigation is limited^15, 43, 44^. Recently, Kravets *et al*^15^ used an ellipsometric spectrometer to probe the sharp phase transitions in plasmonic metamaterials for biosensing. These conventional ellipsometer-like optical readers utilize orthogonally polarized components of the same beam as reference and signal in an oblique reflectance setup. Alternatively, Junesh *et al*^43^ proposed to use an interferometric substrate with an Al mirror layer in order to access the phase information associated with the resonance modes of short-range ordered nanohole arrays in transmission and extracted the phase data from oscillating spectral interference patterns. The common drawback of these techniques is that they cannot provide spatial information, which is essential for high-throughput multiplexed biosensing. Importantly, despite their advantages, these traditional spectrometer-based optical setups are complex and bulky, thus limiting the use of phase interrogation, especially for POC applications.

Differential interference contrast microscope, which uses sheared beams, is a phase-reading technique that allows two-dimensional phase mapping on a limited field of view (FOV), but its precision relies on critical alignment of lens and Wollaston–Nomarski angle-shearing prism^45^. Similarly, interferometric scattering microscopy is a label-free platform with high phase sensitivity that suffers from limited FOV due to the use of high numerical aperture objectives^46, 47^. Recently, a novel phase-interrogating large FOV interferometric microscope was introduced, offering ultrasensitive axial topographic resolution in a lens-free and compact form^48^. Phase detection was achieved by first shearing the incoming light into signal and reference optical beams, and then interferometrically combining them in a collinear common-path geometry. The corresponding beams are quasi-overlapped and thus subjected to similar environmental instabilities, leading to reduced noise. Measurements on transparent substrates showed the potential of the device, but the demonstrated sensitivity was not sufficient for biosensing applications.

In this paper, we present a new plasmonic phase-sensitive detection platform for measuring protein biomarker microarrays with high throughput. It consists of a specifically designed metal nanostructured chip that enhances phase effects of near-field light–matter interactions, which are then detected at the far field using a large FOV lens-free differential interference contrast microarray (LIM) imager. The plasmonic interaction increases the phase sensitivity of the previously demonstrated^48^ LIM setup by more than one order of magnitude, which is crucial for efficient biomarker detection.

**Figure 1:**
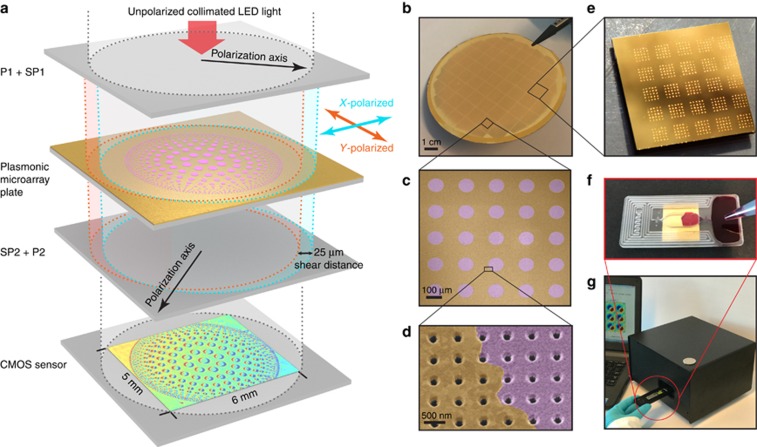
Large FOV interferometric microarray imager (LIM) and experimental setup. (**a**) Collinear optical light-path configuration of the LIM setup. Collimated LED light beam is polarized (P1) and then sheared by a SP (SP1). This generates quasi-spatially overlapped and orthogonally polarized light beams that traverse the plasmonic microarray plate and are subsequently recombined using a second SP (SP2) and interfered by a second polarizer (P2). The interferogram is finally imaged by the CMOS sensor. The image shown on the schematic CMOS sensor is a real measured interferogram of a 10 nm-thin silica (SiO_2_) pattern on a plasmonic chip. (**b**) Photograph of a wafer comprising 1 cm × 1 cm plasmonic Au-NHA chips fabricated using high-throughput, wafer-scale nanofabrication tools. (**c**, **d**) Artificially colored scanning electron microscopy images of 10 nm thin silica microarrays on uniformly patterned plasmonic Au-NHAs. (**e**) Photograph of a plasmonic chip with 200 pl volume protein droplet microarrays formed using low-volume liquid dispensing tool. (**f**) Disposable capillarity-based microfluidic platform assembled on the plasmonic microarray plate. (**g**) Portable interferometric microarray imager operated through an interface running on an ordinary personal computer.

Figure 1a depicts the large-area plasmonic microarray plate, sandwiched between two polarizer (P), P1 and P2, and Savart plate (SP), SP1 and SP2, pairs. The output from a collimated LED source is split by SP1 into two orthogonally polarized beams sheared with respect to each other (shear distance is 25 μm) that traverse the microarray plate. The two sheared beams are recombined by SP2 and interfered by P2, which is orthogonal to P1. This optical design allows for the projection of minute topographical changes, enhanced by the plasmonic interaction, onto a CMOS image sensor (~30 mm^2^). Consequently, any phase difference between the two beams are mapped over the entire sensor chip. In Figure 1a, we show a CMOS image of the experimentally measured optical path difference (OPD) map from a plasmonic chip patterned with arrays of 10 nm thin silica (SiO_2_) layer. In order to emphasize the flexibility of our method in recognizing microspots with different diameters and interspot distances, we designed this pattern where the diameter of the spots decrease from center to edge (350–50 μm), as well as the spacings in between them (min 25 μm). The corresponding OPD contrast from the microarrayed silica spots is clearly observed (Supplementary Fig. 4S). The OPD maps are computed using multiple interferograms collected at different SP1 tilt positions controlled by a stepper motor and applying a phase-shifting interferometry method.

For the first time, we exploit the sharp phase transitions at the resonances of plasmonic gold nanohole arrays (Au-NHAs)^49^, instead of the most commonly used amplitude-sensitive approach and demonstrate high-throughput biosensing using the LIM imager. The microarray plate, consisting of uniformly nanostructured Au-NHAs over the entire sensor surface, is fabricated on robust glass wafers using high-throughput and low-cost deep-ultraviolet lithography. Such scalable and robust manufacturing techniques are necessary for disposable and affordable biosensors. Figure 1b shows a 4″ wafer comprising 50 Au-NHA chips (1 cm × 1 cm). To characterize the proposed plasmonic phase-sensitive detection platform, we first probed it using dielectric thin films of amorphous silica (Figure 1c and 1d), and showed that atomically thin (angstrom-level) topographical changes in a microarray setting with potentially millions of protein biomarker spots can be detected. Subsequently, to validate the platform as a biosensor, we created microarrays of protein solution droplets on the plasmonic chips via low-volume liquid dispensers (Figure 1e). The bioassays were carried out using a capillarity-based disposable microfluidic platform, which enables on-chip, pumpless fluidic manipulation (Figure 1f). We experimentally extracted the OPD sensitivity of our phase-sensitive reader as 9000 nm per refractive index unit (RIU) and the minimum detectable refractive index change as 5.7 × 10^−4^ RIU, which is an order of magnitude higher than the intensity-based imaging system values reported by Cetin *et al*^28^. Moreover, the LIM imager is highly integrated (portable) and built with low-cost off-the-shelf consumer electronic and optic products, such as CMOS image sensors and LEDs (Figure 1g), and it can also be operated by a standard personal computer through a simple user interface. The readout time for a single measurement is 30 s. All of these features are essential for practical and rapid POC biosensing.

### Detection principle

**Figure 2:**
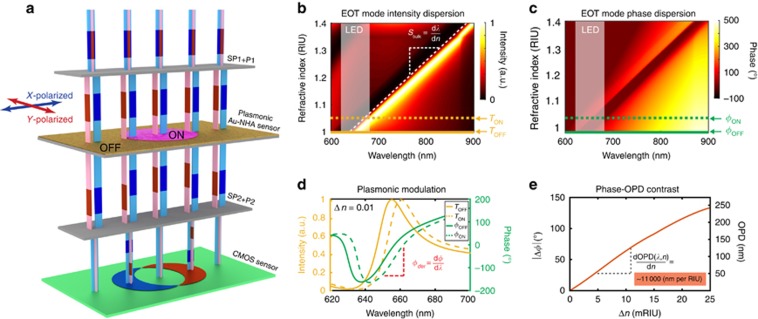
Phase interrogation principle of LIM on plasmonic substrates. (**a**) Orthogonally polarized and symmetrically sheared, that is, partially overlapped, beams (red and blue columns) are both intensity and phase modulated upon traversing the Au-NHA sensor, due to the plasmonic mode coupling. The plasmonic phase and intensity modulation show spatial difference on the microarray spots (ON) with respect to the bare plasmonic surface (OFF). When the light beams are recombined (that is, the shear is removed), they create fringe patterns indicated by blue and red regions on the CMOS sensor due to phase differences induced by the distinct ON and OFF plasmonic phase modulation. (**b**, **c**) Numerically computed transmission intensity and phase dispersion of the EOT mode plotted as a function of the refractive index (RI) of the top media. The redshift of the mode associated with the RI increase can be observed both in the transmission intensity and phase plots (see contrast in the color maps). The bulk sensitivity (*S*_bulk_) of the Au-NHAs (period 600 nm and diameter=200 nm) is calculated as 615 nm per RIU. The LED source, with peak wavelength *λ*_peak_=656 nm, is also indicated on the plot. (**d**) Representative intensity and phase modulation spectra associated with the ON and OFF regions with a RI difference (Δ*n*) of 0.01. The phase derivative (*ϕ*_der_) corresponding to the EOT peak, which is a significant parameter in phase interrogation, is marked on the plot. (**e**) Phase contrast (Δ*ϕ*) and the corresponding OPD 
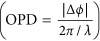
 between the ON and OFF regions as a function of Δ*n* are calculated at the EOT resonance wavelength (*λ*_EOT_) of the bare sensor. The refractometric LIM sensitivity can be numerically calculated from the slope of the curve.

The plasmonic Au-NHAs retain the unique extraordinary transmission (EOT) phenomenon induced by the interplay of two resonance coupling mechanisms in a classical asymmetric Fano-type spectral line profile^22, 50^. Perpendicularly incident light generates in-plane surface plasmon polariton sub-radiant modes satisfying the Bragg’s coupling condition through wavevector matching of the grating’s momentum. The sharp dark mode strongly couples to the subwavelength holes in the Au film and generates a bright radiant mode that scatters light into the free space. These destructive and constructive near-field interactions are strongly dispersive and manifest themselves in the far-field intensity spectrum as multiple dips and peaks that constitute different modes associated with either the supporting substrate or the top medium. It is imperative for plasmonic biosensing that the sensing mode be spectrally isolated from other modes as the refractive index of the monitored medium changes^51, 52^. A well-designed mode allows sensing with different background media, such as air (*n*_air_=1) or water (*n*_water_=1.33), and ensures high sensitivity over a wide dynamic range. In this work, we use the primary (−1, 0) EOT sensing mode of the Au-NHA sensor (hole diameter=200 nm and period=600 nm) and suppress the other substrate modes by increasing the thickness of the Ti adhesion layer (10 nm) between the glass substrate and the nanostructured Au film. Figure 2b shows the numerically computed intensity dispersion curve of the primary (−1, 0) EOT sensing mode, which is spectrally well isolated. The slope of this continuous EOT mode allows us to calculate the conventional bulk refractive index sensitivity in the visible-to-near-infrared range as 615 nm per RIU (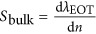, where *λ*_EOT_ is the transmission peak wavelength and *n* is the refractive index of the top medium), which is in line with the experimental findings reported in the literature^22, 51^. In comparison to the intensity, the phase response of the plasmonic resonances has not been widely explored thus far. The incident light that couples to the plasmonic surface experiences temporal retardation at the resonance modes, resulting in sharp phase transitions in the far field^53^, which can be exploited for sensing the refractive index variations on sensor surface. Figure 2c shows that phase dispersion curve exhibits the same bulk sensitivity calculated on the intensity dispersion curve of Figure 2b.

In order to access the phase information from the plasmonic Au-NHA sensor, we use the LIM imager introduced in Terborg *et al*^48^. This imager offers precision and stability over a large FOV by interfering sheared common-path collinear beams. The orthogonally polarized and sheared beams, shown in Figure 2a as red and blue columns, traverse the plasmonic sensor where they are both intensity and phase modulated. As the Au-NHA plasmonic resonances are polarization-independent^28^, the modulation functions vary only spatially depending on whether the material to be detected is present or absent at a given region on the sensor surface (In Figure 2a, the pink region represents a microarray element to be detected). Specifically, the beam that traverse the microarray element is referred to the signal and is modulated by the ON functions; whereas, the beam that traverse the bare sensor is referred to the reference and is modulated by the OFF functions, as shown in Figure 2d. Note that the presence of material on the sensor surface induces a redshift in the spectral positions of both the intensity and phase modulation functions. When the beams reach the second SP and polarizer, they are recombined and interfered, and the topography of the microarray pattern on the sensor surface is projected as interference contrast fringes on the CMOS sensor. In Figure 2a, the green pixels of the CMOS sensor correspond to regions where the sheared beams go through identical optical paths, and therefore yield zero intensity after interference. Whereas the blue and red pixels correspond to the regions where the beams are modulated by different phase transmission functions (*ϕ*_ON_ and *ϕ*_OFF_), resulting in a net intensity after interference. In contrast to transparent substrates (that is, no plasmonic structure) where the phase difference depends only on the thickness of the material and its refractive index contrast with the medium, in our system the phase difference is amplified by the plasmonic phase function, which results in a sensitivity increase.

Unlike the previous intensity-based plasmonic biosensors, which rely only on the spectral position of the EOT intensity peak, our methodology uses the OPD between the signal and reference beams (OPD=Δ*ϕ*/*k*, where *k*=*λ*_EOT_/2*π* is the wavevector and Δ*ϕ*=|*ϕ*_ON_−*ϕ*_OFF_|). In our interferometric reader, this phase contrast value (Δ*ϕ*) is extracted from multiple images recorded at different phase bias using the computational phase-shifting interferometry technique^48^. Figure 2e shows the numerically computed phase contrast Δ*ϕ* (left axis) and OPD (right axis) as functions of the refractive index difference (Δ*n*=*n*_material_−*n*_medium_). The sensitivity of our system can also be analytically stated as follows:


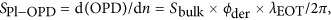


where *ϕ*_der_=d*ϕ*/d*λ*=10 [deg nm^−1^] at the EOT peak (indicated in Figure 2d). The OPD sensitivity can, therefore, be numerically estimated as 1.1 × 10^4^ nm per RIU over a dynamic range of 0.025 RIU, which is sufficient for biosensing applications. In our system, this dynamic range is mainly defined by the spectral shape of the plasmonic mode, which correlates to the derivative of the phase function (*ϕ*_der_), as well as the spectrum of the illumination source. Details on the plasmonic phase sensitivity (*S*_Pl-OPD_) derivation are presented in the Supplementary Information.

## Materials and methods

### Fabrication of Au-NHA plasmonic chips

Au-NHA chips are manufactured using wafer-scale, high-throughput nanofabrication techniques on a robust transparent substrate in a single lithography step. First, Radio Corporation of America-cleaned 4-inch fused silica wafers are coated with Ti/Au (10/120 nm) using an e-beam evaporator (Alliance-Concept EVA 760, Cran Gevrier, France). The 10 nm-thick Ti layer not only enables the Au-adhesion on the glass substrate but also suppresses the irrelevant surface modes induced by the glass–Au interface. Therefore, only the (1, 0) EOT peak can be monitored through a wide spectral interval, which is crucial for wide dynamic sensing range. Next, the nanohole arrays (200-nm diameter and 600-nm period) are patterned using a 248 nm deep-ultraviolet stepper (ASML PAS 5500/300 DUV, Veldhoven, Netherlands). After resist development, the nanohole arrays are transferred into the Ti/Au layer using an ion beam etching tool (Oxford Instruments PlasmaLab 300 IBE, Abingdon, UK). The resist on the surface is removed using oxygen plasma cleaning. Finally, after coating with a layer of photoresist to protect the sensor surface, the 4-inch wafer is diced into 1 cm × 1 cm chips (Figure 1). The resist layer on the chips is removed by a remover solvent followed by an oxygen plasma exposure to ensure a uniformly clean sensor surface.

### SiO_2_ microarray patterning

The Au-NHA and the transparent fused silica chips are post-patterned to form SiO_2_ thin-film microarrays. First, a single-step lithography is performed using a bi-layer photoresist stack (top: AZ1512 HS, bottom: LOR; MicroChemicals, Ulm, Germany). After resist development, various thin SiO_2_ layers are evaporated on both Au-NHA chips and their corresponding transparent controls using an e-beam evaporator (Alliance-Concept EVA 760). Finally, a lift-off process in resist remover solvent is applied, leaving a microarray of circular SiO_2_ thin-film patterns (Figure 1a).

### Spectroscopic chip validation

The fabricated Au-NHA chips are spectrally characterized using an imaging spectrophotometer (IsoPlane 320, Princeton Instruments, Trenton, NJ, USA), which is directly coupled to the outlet of a Nikon (Tokyo, Japan) Eclipse-Ti inverted microscope. The transmission spectra of the Au-NHA chips are recorded under normal white light illumination and examined for the EOT peak position and bandwidth validation.

### Immobilizing bioreceptors on the sensor surface

To enable the biorecognition assays, the gold surface is modified with detection proteins using noncovalent physicochemical adsorption. First, protein A/G (recombinant fusion protein with protein A- and protein G-binding sites) solution (500 μg ml^−1^) in 10 mM acetate buffer (pH 4) with 0.5% glycerol is deposited on the clean Au sensor in a microarray format. A non-contact, low-volume liquid dispenser (sciFLEXARRAYER S3, Scienion, Dortmund, Germany) is used to create microarrays of protein A/G droplets (150 pl) with 100 μm diameter and 250-μm period. After 1 h incubation, the chips are washed in phosphate-buffered saline (PBS, pH 7.4) for 5 min under constant agitation to remove excess proteins. The remaining area on the bare Au surface is blocked by incubating the whole chip in 1 % v/v bovine serum albumin (BSA) in PBS. Then, the chip is sequentially incubated in mouse IgG solution (Abcam-ab37355, Cambridge, UK, concentration 200 μg ml^−1^) and its matching goat anti-mouse IgG solution (Sigma-Aldrich-M2650, St Louis, MO, USA), each step followed by PBS washing. Wet media measurements are performed in PBS buffer, whereas for dry measurements, chips are rinsed in deionized water to remove the salt residuals and dried with N_2_ after each incubation step.

### Fluidic platform and liquid media measurements

The liquid manipulation on the plasmonic chip is enabled by a disposable microfluidic cartridge (microTEC Ges. für Mikrotechnologie mbH, Duisburg, Germany), fabricated using rapid micro product development technology. The fluid flow is induced by capillary forces without the need of an external pump. The cartridges are mass-produced at low cost from poly (methyl methacrylate) using photo-polymerization. The microfluidic cartridge design (dimensions: 14 mm × 32 mm × 1.01 mm) includes three parts: load chamber; measurement chamber; and waste channel (Figure 1f). The load chamber is open on the top side to access during the liquid insertion. The measurement chamber is open on both sides, to which the surface-functionalized plasmonic chip (bottom) and a glass slide (top) are mechanically sealed. The waste channel is designed to have equal volume (~70 μl) with the measurement chamber and contains two air-release spots, positioned at the beginning and at the end of the channel. Whereas the first air release is initially kept open, the second air release is sealed with a thin membrane facing the top side of the cartridge.

For the liquid media measurements, first, the analyte buffer with the biomarker of interest is inserted into the load chamber. Driven by the pressure equilibrium established with the open first air release, the fluid is passively transferred from the loading well into the measurement chamber by the capillary forces. At this point, the plasmonic microarray plate patterned with capture antibodies is incubated with the analyte solution. After the incubation, the rinsing buffer is added into the load chamber and the second air release is punctured to enable the transfer of analyte solution into the waste channel and perform the final readout.

### Optical reader setup

The optical reader, with dimensions of 23 cm × 21 cm × 15 cm, is composed of three modules: light source; optical assembly; and electronics (Figure 1g).

The light source is an external LED block (Mightex, CA, USA), which comprises four high-power LEDs that are coherently coupled into a single multimode fiber. The LEDs are chosen to spectrally overlap with the Au-NHA transmission peak positions: two in the red wavelength region (625 and 656 nm) for dry and two in the near-infrared (850 and 870 nm) for wet medium measurements.

The optical assembly comprises optical elements that are mounted to facilitate a vertical optical axis. First, the fiber-coupled light is collimated using a parabolic mirror to enable uniform illumination over the FOV. The collimated and coherent light beam passes through the microarray plate mounted on the sample holder, which is sandwiched between two sets of orthogonal P and SP pairs, and terminates at the CMOS sensor (area=4.2 × 5.7 mm^2^). Whereas all optical elements are fixed to the optical column, the top SP holder can be tilted in the *z* direction. A step motor, whose rotation axis is shared by a leveler with uneven radius, is used to push one side of the SP holder to generate precisely controlled tilt angles of up to ±2°.

## Results and discussion

Mass manufacturing of plasmonic biosensors for large FOV interferometric microarray imaging requires highly precise nanofabrication techniques that produce chips with low variability. Specifically, the reliability of our read-out method relies on Au-NHAs that exhibit uniform plasmonic resonance properties over a large sensing field (30 mm^2^). Particularly, the variation of the EOT peak position (*λ*_EOT_) and its bandwidth should be sufficiently small not only within a chip but also between chips fabricated on wafers in different batches. In order to investigate the effects of process variations on the plasmonic resonance properties, we performed a statistical parameter analysis over a sample set of spectral data, collected over 176 chips from four wafers. We found the average EOT peak to be at 656±1.11 nm and the full width half max of the resonance peak to be 25±1.33 nm. Thus, we conclude that the high-throughput and large-scale fabricated plasmonic Au-NHA chips are exceptionally robust and their optical parameters are consistent. Further details on this statistical analysis are given in the Supplementary Information and the fabrication details are presented in the Materials and Methods section.

**Figure 3:**
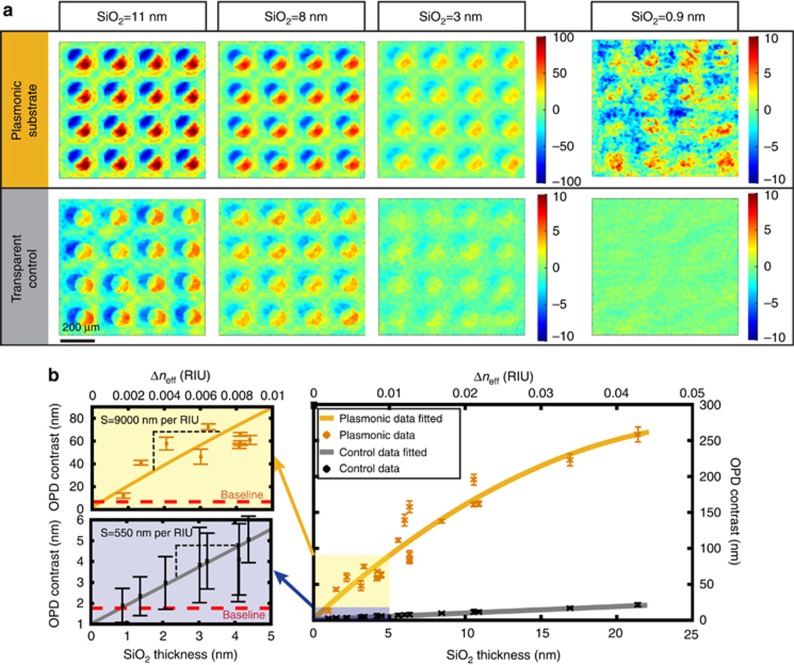
Silica microarray pattern detection with LIM: comparison of plasmonic and transparent substrates. (**a**) Color-coded OPD maps of various microarrayed silica (SiO_2_) thicknesses patterned identically on plasmonic and transparent control substrates. (**b**) OPD contrast data statistically extracted (36 spots considered on each chip) from plasmonic and transparent microarray patterns are shown as a function of silica thickness, with the low thickness range magnified on the left. The top axis indicates the effective refractive index units of the corresponding thin silica films, for convenience. Baseline indicates the average OPD contrast of the bare substrate. Error bars correspond to standard deviation where *N*=36.

To evaluate the microarray detection capability of the phase contrast LIM imager, large microarrays of silica layers with numerous thicknesses were identically patterned on plasmonic Au-NHAs and transparent (control) substrates. For clear visibility, representative OPD maps of 4 × 4 subsets from large microarrays with different silica thicknesses are shown in Figure 3a. With our interferometric method, a circular pattern on the plasmonic sensor is imaged as two crescent shapes (color-coded as red and blue in Figure 3a), each with opposite OPD values. The overall contrast is calculated from the difference between these regions and, therefore, the experimentally acquired OPD values are expected to be twice the numerically estimated ones. The details on the OPD data extraction can be found in the Supplementary Information. Figure 3b shows the average OPD contrast data as a function of the silica thickness (bottom axis) and effective refractive index difference, Δ*n*_eff_ (top axis). Calculation of Δ*n*_eff_ from thin silica films with different layer thicknesses is specified in Supplementary Section. In Figure 3b, each data point represents the average OPD contrast over 36 microarray elements and the baselines correspond to contrasts measured in the absence of any silica layer. We calculated the OPD sensitivity for silica thicknesses below 5 nm from the slope of the fitted curve (see left-most plots in Figure 3b). We experimentally found that the plasmonic sensors exhibit significantly higher (~16 ×) OPD sensitivity (2 × *S*_Pl-OPD-Exp_=9000 nm per RIU) when compared to the transparent controls (2 × *S*_Cl-OPD-Exp_=550 nm per RIU). This is in agreement with our numerical calculations (Supplementary Information), where 2 × *S*_Pl-OPD_ for plasmonic and 2 × *S*_Cl-OPD_ for transparent substrates are estimated as 2.2 × 10^4^ and 570 nm per RIU, respectively.

Plasmonic phase modulation also enables detection of atomically thin layers, which cannot be resolved on transparent substrates, as demonstrated in Figure 3a (SiO_2_=9 Å). In our experimental evaluation using thin dielectric layers, we were limited by the deposition tolerance of the evaporation system, which cannot deposit silica layers thinner than 9 Å. Therefore, as the ultimate detection limit could not be experimentally assessed, we estimated the minimum detectable thickness to be 2.5 Å by interpolating the detection curve toward the background baseline.

**Figure 4:**
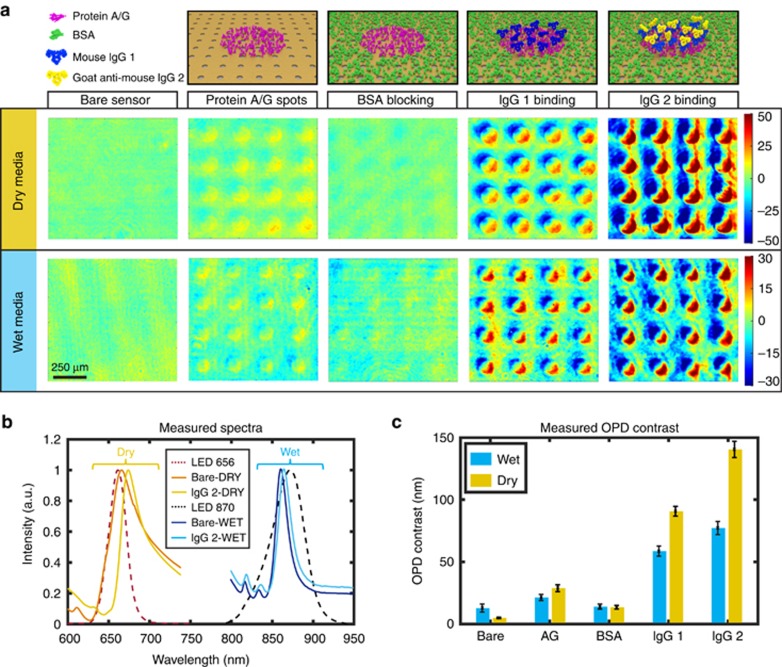
Protein microarray detection with LIM using the plasmonic phase interrogation scheme. (**a**) Color-coded OPD maps of protein microarrays in dry and wet media are presented with corresponding protein stack information on the top row. (**b**) Measured transmission spectra ON and OFF the IgG2 spots in both dry and wet media, as well as the LED illumination spectra. (**c**) OPD contrast data statistically collected (36 spots considered on each chip) at each step of protein layer formation in dry and wet conditions. Error bars show standard deviation where *N*=36.

To evaluate the biosensing performance of our technology, we demonstrate microarray detection of protein monolayer stacks in both dry and wet media. A protein/antibody set with well-known high affinity was used in our experiments. Figure 4a shows the schematic views of protein/antibody layers in their sequential formation and the corresponding representative 4 × 4 microarray OPD map readouts. First, protein A/G solution (0.5 mg ml^−1^) in 150 pl droplets was spotted to define the capture layer of the microarray on bare plasmonic chips. The remaining areas were then blocked with BSA (1% v/v) to form an unreactive thin layer around the protein A/G-covered microspots. Using the affinity of the protein A/G to the immunoglobulins (IgG), we tested the biosensing platform by adding two layers of IgGs. As the topographic contrast is measured with respect to the neighboring areas of the microspots, the BSA layer acts as a negative control for the IgG detection. Figure 4a further depicts the OPD maps that clearly indicate a contrast increase upon the addition of the two subsequent IgG layers.

For illumination, we used two different LEDs with peaks at wavelengths of 656 and 870 nm, which are aligned with the resonance peaks of Au-NHAs in the dry and wet media, respectively. Figure 4b shows the LEDs’ spectra and the measured transmission intensity spectra of Au-NHA sensors using a conventional spectrophotometer coupled to an inverted microscope. The typical spectra associated with the bare and IgG2-coated sensor, in both wet and dry background, are also presented in Figure 4b. As expected, the peak shift is more significant (~9 nm) in dry medium when compared to wet medium (~4 nm), as the refractive index contrast that a biolayer has with air (*n*_air_=1) is higher than that with water (*n*_water_=1.33). This can also be observed in the statistically collected OPD contrast data summarized in the bar plots shown in Figure 4c, which show the average and the s.d. (*N*=36) after each biolayer formation. The magnitude of the OPD contrast associated with each protein layer depends mainly on their different molecular weights (A/G ~50 kDa and IgG ~150 kDa). Additionally, the OPD contrast data acquired from the bare chip 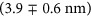 in Figure 4c, together with the experimental OPD sensitivity (9000 nm per RIU), can be used to identify the minimum detectable RIU change (5.7 × 10^−4^ RIU). The results show that our method can be used for bioassays with multiple steps and the sensing data can be obtained in both wet and dry media from a large set of microarray spots.

Moreover, we performed a quantitative analysis and acquired a calibration curve on the third layer of the protein stack, namely IgG2 obtained by incubating the IgG1 microarrays with different concentrations of IgG2 buffer solution. The data were acquired by subtracting the OPD contrast from 36 microarray elements before and after the IgG2 capture (Supplementary Fig. 5S) and it closely follows a saturation curve, which is common in surface sensors due to limited available capture sites. Our preliminary results show that our method can detect a concentration of IgG2 as low as 500 ng ml^−1^ and this limit of detection is an order of magnitude higher than previously reported values for intensity-based biosensor using Au-NHAs^28^. Although this limit of detection is significant, it can be further enhanced, for example, if covalent surface functionalization methods, such as the traditional thiol chemistry on Au surface, are used instead of the physicochemical adsorption of capture proteins. Particularly, to detect low-concentration analytes in real biological fluids, such as serum, a robust surface functionalization together with high-affinity capture agents are essential.

We also performed the protein detection experiments discussed above on transparent glass plates with the same LIM imager in similar experimental conditions reported before^48^, but the microarrays could not be detected. Note that the Au-NHA substrates only enhance the phase signal when material to be detected is within the reach of plasmonically enhanced surface fields (<100 nm), contrary to the transparent substrates where the total volume between the polarizers are probed. Therefore, plasmonic substrates not only enhance the biosensing signal from extremely small volumes near the surface but also suppress the irrelevant fringes from the background media. This emphasizes the importance of plasmonic phase enhancement for biosensing applications.

Our biosensing platform enables simultaneous multiplexed data collection from large microarrays thanks to its wide FOV, without the need for image stitching or stage moving. In this work, we also used computational diffraction correction methods^48^ to increase the lateral resolution of the lens-free reader. In the LIM system, the lateral image resolution and the shear distance of the SPs delimit the density of the microarray. In our current reader (shear distance=25 μm), we can simultaneously monitor thousands of microarray elements in a 30 mm^2^ FOV, as shown in the sample pattern in Figure 1a. Our calculations show that hardware and software improvements, such as birefringence elements with smaller shear distance, detectors with higher pixel density and improved diffraction correcting algorithms, can further reduce the lateral resolution down to the CMOS pixel size, that is, 4 μm. This would potentially allow simultaneous access to over a million biomarkers thus enabling extraordinary multiplexing capability. Moreover, the integrated disposable fluidic chip enables simple analyte insertion and the straightforward user interface eliminates the need for trained staff for on-site measurements. Additionally, the interferometric imaging method extracts the sensing signal by comparing it to reference signal from neighboring areas around the microspot. This is a crucial property for biosensing as nonspecific binding signals are inherently suppressed. Finally, while we only show the end-point measurements, our platform can also perform kinetic measurements when a fluid insertion pump together with a compatible microfluidic flow cell is assembled to the chip.

## Conclusions

In this work, we proposed and demonstrated a novel platform for high-throughput and effective biosensing that exploits plasmonically enhanced phase modulation in a compact, robust and portable interferometric imager. Plasmonic phase modulation in a collinear optical setup produces two-dimensional fringe maps on a large-area consumer electronic CMOS imager of atomic-scale topographical changes, without the need for intricate and expensive optical equipment. Moreover, we achieved low-cost and robust manufacturing of the nanostructured plasmonic sensor chips using deep-ultraviolet lithography technology capable of uniformly nanopatterning an entire 4″ wafer. Increasing the wafer size, or utilizing other wafer-scale nanofabrication methods^54^, such as interference lithography, could further reduce the cost per chip. We achieved high sensitivity in this integrated nanophotonic system by leveraging the sharp transmission resonance of nanostructured plasmonic Au-NHA sensors. The exceptional high-content microarray multiplexing can provide access to extremely large data sets of biomolecular information acquired from small quantities of biological samples, both saving time and reducing cost of clinical testing. This disruptive technology can exploit data science methodologies to cross-correlate the ‘big data’ that is readily accessible from patients to transform the practice of medical diagnostics and enable remote health monitoring. In summary, our platform can have a significant impact in POC rapid detection of life-threatening diseases and advance next-generation health care, while opening new avenues for exploration of nontrivial phase response of nanophotonic systems for sensing applications.

## Author contributions

FY, RT, JP, VP and HA designed the experiments and conceived this study. VP proposed the EU Horizon 2020 RAIS project. FY performed the numerical computations. FY and YJ designed and fabricated the plasmonic chips. FY and AB performed the bioexperiments. FY, RT, JP and AB developed the code for data processing and prepared the data. FY, VP and HA wrote the paper with contributions from all authors.
