# Supplementary material for: Phase-sensitive plasmonic biosensor using a portable and large field-of-view interferometric microarray imager
Source: Light Sci Appl. 2018 Feb 23;7:17152–. doi: 10.1038/lsa.2017.152 (PMC6060062; doi:10.1038/lsa.2017.152)
Supplement: Supplementary Information [file lsa2017152x1.pdf]

Supplementary information

Phase-sensitive plasmonic biosensor using a portable and large field-of-view interferometric microarray imager

Filiz Yesilkoy[1], Roland A Terborg[2], Josselin Pello[2], Alexander A Belushkin[1], Yasaman Jahani[1], Valerio Pruneri[2,3] and Hatice Altug[1ss]

[1]*Institute of BioEngineering, École Polytechnique Fédérale de Lausanne, CH-1015 Lausanne, Switzerland*

[2]*ICFO—Institut de Ciències Fotòniques, The Barcelona Institute of Science and Technology, 08860 Castelldefels (Barcelona), Spain*

[3]*ICREA—Institució Catalana de Recerca i Estudis Avançats, 08010 Barcelona, Spain*

Phase sensitivity derivation of plasmonically enhanced interferometric interrogation

In this section we present the effect of the plasmonic phase and intensity modulations on the light that propagates through the optical elements of our interferometric imager, and we derive the phase sensitivity expression as a function of plasmonic resonance parameters. Moreover, we estimate the phase sensitivity of both the plasmonic and the control transparent substrates by evaluating the derived expression with the numerically computed plasmonic parameters.

The following notation is used in this section:

λ	Wavelength
t	Time
c	Speed of light in vacuum
ω	Angular frequency, $2\pi c/\lambda$
k	Wave vector, $2\pi/\lambda$
n	Refractive index of the material to be detected
E_0	Amplitude of the electric field component of electromagnetic wave
I	Intensity
α	Phase bias
l_{SiO_2}	Silica layer thickness

The intensity of the incident light from the LED source on the plasmonic sensor surface is given by:

$I_{inc}(\lambda) = 2E_0^2(\lambda).$ (1)

The wave equations of the collimated, *X* and *Y* polarized and sheared beams incident on the plasmonic surface can be represented in the plane wave form:

$E_x(\lambda, t) = E_0(\lambda)e^{i(kz-\omega t)}$ (2)

$E_y(\lambda, t) = E_0(\lambda)e^{i(kz-\omega t)}.$ (3)

The transmitted light through the plasmonic Au-NHA surface is both intensity and phase modulated. The intensity and phase modulation functions vary spatially depending on the effective refractive index change associated with the material to be detected. The modulation functions are distinguished by "*ON*" and "*OFF*" tags, corresponding to the light transmitted through the patterned area (the sensing region) and bare area (the reference region), respectively. While the modulation functions of the reference region are determined by the bulk refractive index of the top media only, the sensing region functions also depend on the refractive index change that the material induces. Moreover, the modulation functions of Au-NHAs are polarization independent due to Au-NHAs' symmetric geometry.

The intensity modulation functions (also shown in Fig.2d) are given by:

$T_{x,ON} = T_{y,ON} = T_{ON}(\lambda, n)$ (4)

$T_{x,OFF} = T_{y,OFF} = T_{OFF}(\lambda),$ (5)

and the phase modulation functions (also shown in Fig.2d) are given by:

$\phi_{x,ON} = \phi_{y,ON} = \phi_{ON}(\lambda, n)$ (6)

$\phi_{x,OFF} = \phi_{y,OFF} = \phi_{OFF}(\lambda).$ (7)

The wave equations of the transmitted *X* and *Y* polarized beams, both phase and intensity modulated, can be calculated using equations 2-7:

$\boldsymbol{E_{x,ON}(\lambda, t, n)} = \boldsymbol{E_{y,ON}(\lambda, t, n)} = \sqrt{T_{ON}(\lambda, n)}\, E_0(\lambda)e^{i(kz-\omega t+\phi_{ON}(\lambda,n))}$ (8)

$\boldsymbol{E_{x,OFF}(\lambda, t)} = \boldsymbol{E_{y,OFF}(\lambda, t)} = \sqrt{T_{OFF}(\lambda)}\, E_0(\lambda)e^{i(kz-\omega t+\phi_{OFF}(\lambda))}.$ (9)

An interferogram is formed on the image sensor, once the light traverses the second savart plate and the polarizer (with polarization axis orthogonal to the first polarizer). A typical interferogram in Figure 1S shows four spatial regions of different intensities.

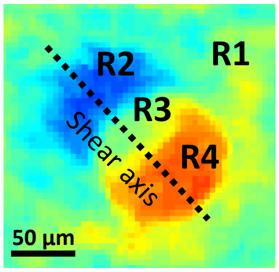

Figure 1S: A typical optical path difference map of a silica circular spot measured on plasmonic Au nanohole array showing four regions corresponding to the double crescent that forms due to the interference of the sheared beams.

$$\boldsymbol{I_{R1}(\lambda)} = \left|E_{x,OFF}(\lambda,t) - E_{y,OFF}(\lambda,t)\right|^2 = 0 \qquad (10)$$

$$\boldsymbol{I_{R3}(\lambda)} = \left|E_{x,ON}(\lambda,t,n) - E_{y,ON}(\lambda,t,n)\right|^2 = 0 \qquad (11)$$

$$\boldsymbol{I_{R2}(\lambda,n)} = \boldsymbol{I_{R4}(\lambda,n)} = \left|E_{x,ON}(\lambda,t,n) - E_{y,OFF}(\lambda,t)\right|^2$$

$$= \left|\sqrt{T_{ON}(\lambda,n)}\,E_0(\lambda)e^{i(kz-\omega t+\phi_{ON}(\lambda,n))} - \sqrt{T_{OFF}(\lambda)}\,E_0(\lambda)e^{i(kz-\omega t+\phi_{OFF}(\lambda))}\right|^2$$

$$= E_0^2(\lambda)\left(\sqrt{T_{ON}(\lambda,n)}\,e^{i\phi_{ON}(\lambda,n)} - \sqrt{T_{OFF}(\lambda)}\,e^{i\phi_{OFF}(\lambda)}\right)\left(\sqrt{T_{ON}(\lambda,n)}\,e^{-i\phi_{ON}(\lambda,n)} - \sqrt{T_{OFF}(\lambda)}\,e^{-i\phi_{OFF}(\lambda)}\right)$$

$$= E_0^2(\lambda)\left[T_{ON}(\lambda,n) + T_{OFF}(\lambda) - \sqrt{T_{ON}(\lambda,n)T_{OFF}(\lambda)}\left(e^{i(\phi_{ON}(\lambda,n)-\phi_{OFF}(\lambda))} + e^{-i(\phi_{ON}(\lambda,n)-\phi_{OFF}(\lambda))}\right)\right]$$

$$= \frac{I_{inc}(\lambda)}{2}\left(T_{ON}(\lambda,n) + T_{OFF}(\lambda) - 2\sqrt{T_{ON}(\lambda,n)T_{OFF}(\lambda)}\cos(\Delta\phi(\lambda,n))\right), \qquad (12)$$

where the phase shift function between the signal and the reference is:

$$\boldsymbol{\Delta\phi(\lambda,n)} = \phi_{ON}(\lambda,n) - \phi_{OFF}(\lambda). \qquad (13)$$

Multiple image acquisitions (30 in our case) at different tilt positions of the top savart plate introduce a phase bias parameter (α), which is only added to the X-polarized component of the beam, and modulates the intensities in the R_2 and R_4 regions (I_{R2} =I_{R4}):

$$\boldsymbol{I_{R4}(\lambda,n,\alpha)} = \frac{I_{inc}(\lambda)}{2}\left(T_{ON}(\lambda,n) + T_{OFF}(\lambda) - 2\sqrt{T_{ON}(\lambda,n)T_{OFF}(\lambda)}\cos(\Delta\phi(\lambda,n)+\alpha)\right) \qquad (14)$$

Using the intensity variations in a series of interferograms taken at different values of α, where $0 \leq \alpha \leq 2\pi$, the phase shift function, $\Delta\phi(\lambda,n)$, can be computed. This method is called phase-shifting interferometry (PSI), and is detailed in Terborg et. al[1].

The phase shift value is then converted into optical path difference (OPD), in nm units, to convey a physical meaning using the relationship below:

$$\boldsymbol{OPD(\lambda,n)} = \frac{\Delta\phi(\lambda,n)}{\frac{2\pi}{\lambda}}. \qquad (15)$$

The OPD sensitivity of the plasmonic chips on the LIM system then can be defined as:

$$\boldsymbol{S_{Pl-OPD}} = \frac{dOPD(\lambda,n)}{dn} = \frac{d(\Delta\phi(\lambda,n))}{dn} * \frac{\lambda}{2\pi} = \frac{d\left(\phi_{ON}(\lambda,n)-\phi_{OFF}(\lambda)\right)}{dn} * \frac{\lambda}{2\pi} = \frac{d(\phi_{ON}(\lambda,n))}{dn} * \frac{\lambda}{2\pi} \quad (16)$$

Note that the transmission phase and intensity spectra only shift to higher wavelengths as n increases without experiencing any change in shape. The rate of this red-shift is defined as bulk sensitivity, S_{bulk}. Therefore, $\phi_{ON}(\lambda, n)$ can be written in terms of $\phi_{OFF}(\lambda, n)$ as follows:

$$\boldsymbol{\phi_{ON}(\lambda, n)} = \phi_{OFF}(\lambda - S_{bulk}(n - n_o)). \quad (17)$$

Let $\boldsymbol{u} = \lambda - S_{bulk}(n - n_o)$ and we have:

$$\boldsymbol{S_{Pl-OPD}} = \frac{d(\phi_{OFF}(u))}{dn} * \frac{\lambda}{2\pi} = \frac{d\left(\phi_{OFF}(u)\right)}{du} * \frac{du}{dn} * \frac{\lambda}{2\pi} = -\frac{\lambda}{2\pi} * S_{bulk} * \phi_{der}, \quad (18)$$

where we define a phase derivative parameter: $\phi_{der} = \frac{d\phi_{OFF}(u)}{du}$. (19)

The phase sensitivity can then be computed at the extraordinary transmission (EOT) peak wavelength, λ_{EOT}, which dominates the phase information measured by the image sensor by providing the largest number of photons. Therefore,

$$\boldsymbol{S_{Pl-OPD}}|_{\boldsymbol{\lambda=\lambda_{EOT}}} = -\frac{\lambda_{EOT}}{2\pi} * S_{bulk} * \phi_{der}|_{\lambda=\lambda_{EOT}} \quad (20)$$

Given that in our plasmonic Au-NHA system,
$S_{bulk} \sim 615 \left[\frac{nm}{RIU}\right], \phi_{der}|_{\lambda=\lambda_{EOT}} \sim 10 \left[\frac{degrees}{nm}\right]$, $\lambda_{EOT} \sim 656\ [nm]$ and using the equation 19, we can estimate the phase sensitivity of the Au-NHA plasmonic system for small refractive index variations on the bare sensor around EOT peak in air medium ($n_{air} = 1$) as:

$$\boldsymbol{S_{Pl-OPD}} = \mathbf{11000}\ [\boldsymbol{nm/RIU}] \quad (21)$$

On the other hand, the phase sensitivity for silica layers ($n_{SiO_2} = 1.45$) as a function of its thickness (l_{SiO_2}) on a transparent control substrate with air background can be stated as:

$$\boldsymbol{S_{Cl-OPD}} = \frac{dOPD(n)}{dn} = \frac{d\left(l_{SiO_2} * (n_{SiO_2} - n_{air})\right)}{dl_{SiO_2}} * \frac{dl_{SiO_2}}{dn} = 0.45 * \frac{dl_{SiO_2}}{dn}, \quad (22)$$

where $\boldsymbol{OPD(n)} = l_{SiO_2} * \left(n_{SiO_2} - n_{air}\right)$. (23)

In order to state the OPD sensitivity in conventional RIU units, we calculated the effective bulk refractive index change (Δn_{eff}) that a thin silica layer creates with air background. A silica film thickness to Δn_{eff} conversion relation is calculated using numerically computed Au-NHA bulk sensitivity and the silica thin film induced λ_{EOT} shift as: $\frac{dl_{SiO_2}}{dn} = 521\ [nm/RIU]$ (see Figure 2S).

After rewriting equation 22, the phase sensitivity on transparent substrates becomes:

$$\boldsymbol{S_{Cl-OPD}} = \frac{dOPD(n)}{dn} = \frac{dOPD(n)}{dl_{SiO_2}} * \frac{dl_{SiO_2}}{dn} = 0.45 * 521 = \mathbf{234.45}\ [\boldsymbol{nm/RIU}] \quad (24)$$

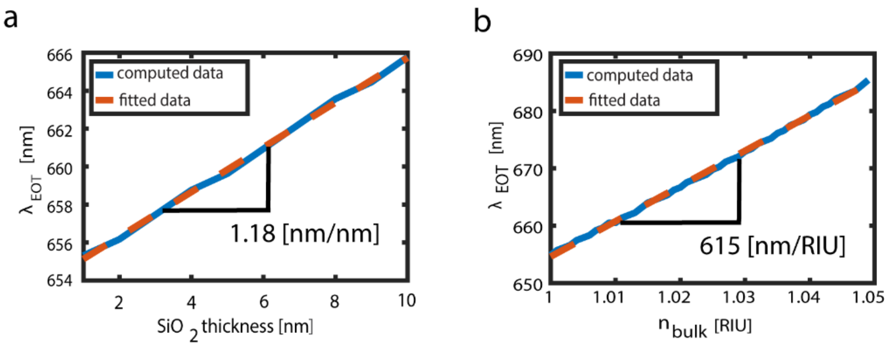

Figure 2S: Au-NHA computed EOT peak sensitivity to a) varying silica layer thickness b) bulk refractive index change.

OPD contrast statistical data extraction

For statistical evaluation, OPD contrast data is extracted from large-area OPD maps using a graphical user interface (GUI), which was custom designed to handle microarrays with different geometric parameters (see Figure 3S). The OPD map, where each pixel represents 4.4 x 4.4 μm^2 area, is color scaled to increase visibility and the array to be considered is manually chosen on the GUI. After defining the grid parameters, unit cells, where a single microspot resides, are identified. Within each unit cell, the highest and the lowest valued “N” pixels are averaged and their difference is taken as “OPD contrast”. The number of pixels (N) considered for the contrast computation depends on the microarray element dimensions (5% of the spot area) in order to keep consistency among different microarrays.

Using a set of interferograms, the computational OPD map generation results in a twin image (see Figure 1S), where the regions R2 and R4 have similar magnitude but opposite signed OPD values. Since we compute the OPD contrast using both of these regions, the experimentally acquired OPD contrast is about twice the original numerical estimate. Thus the numerically estimated sensitivities become $\mathbf{2} \times \boldsymbol{S_{Pl-OPD}} \boldsymbol{\sim} \mathbf{22000}\ [\boldsymbol{nm/RIU}]$ and $2 \times \boldsymbol{S_{Cl-OPD}} \sim \mathbf{570}\ [\boldsymbol{nm/RIU}]$ for plasmonic and transparent substrates, respectively.

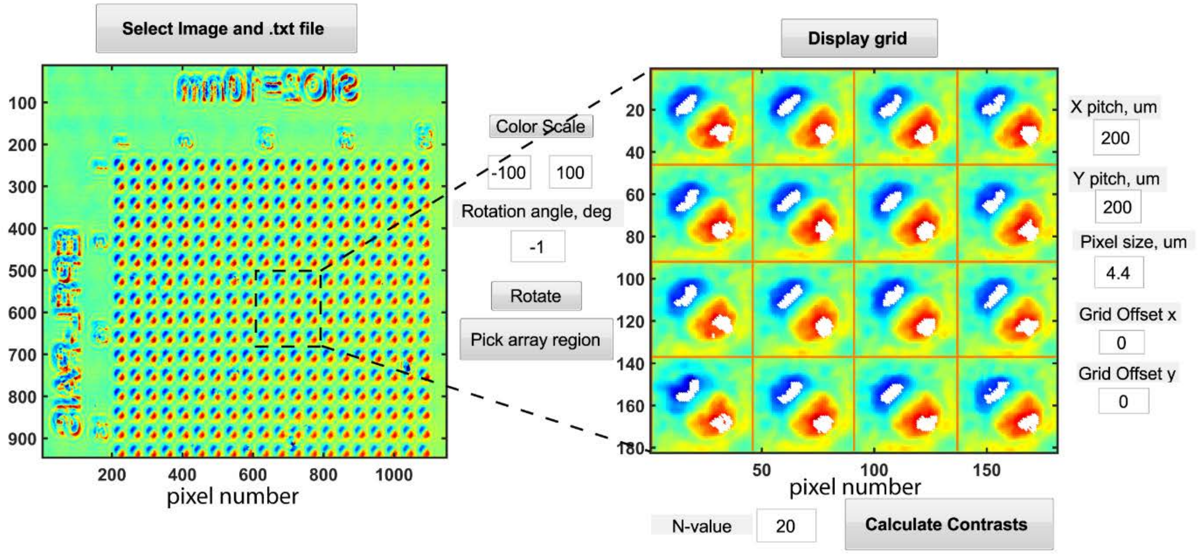

Figure 3S: Graphical user interface used to extract the OPD contrast statistical information over large arrays. The white pixels on the selected array (right) represents the highest and the lowest valued pixels that are considered for the OPD contrast evaluation.

Demonstration of large micro array OPD maps

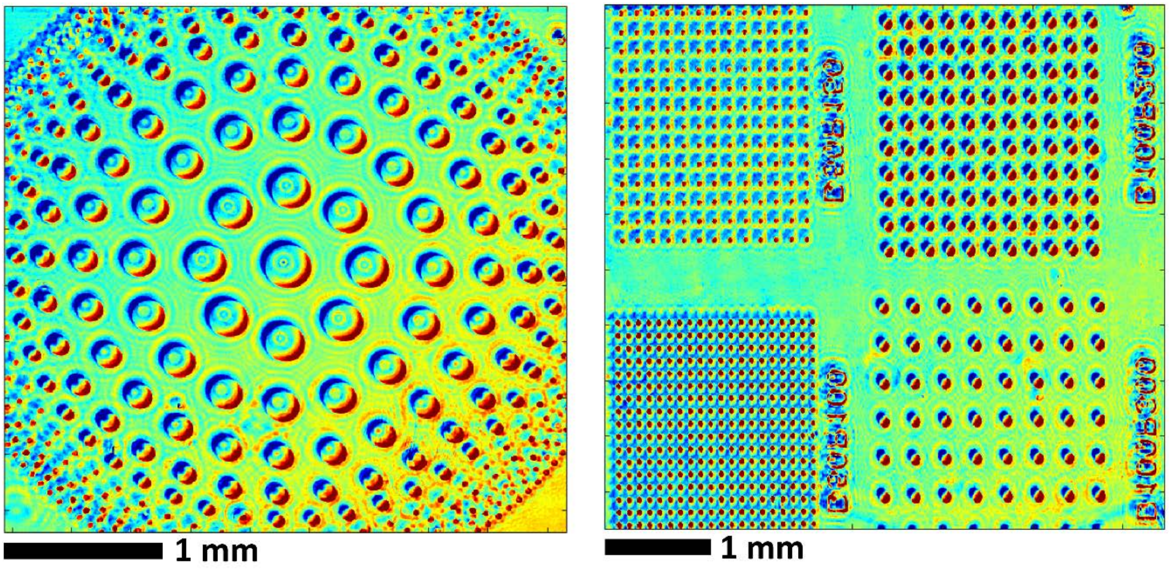

Figure 4S: Large area OPD maps of silica layer (thickness= 10 nm) microarrays on plasmonic sensors. Left: The image shown on the schematic CMOS sensor in Figure 1a. Various microspot sizes (diameter=350-50 µm) and spacings (min 25 µm) is clearly observed. Right: Four different microarrays with dimensions 100/200, 100/300, 50/100, 50/150 (diameter/period in µm) are shown.

Quantitative Biosensing of IgG 2

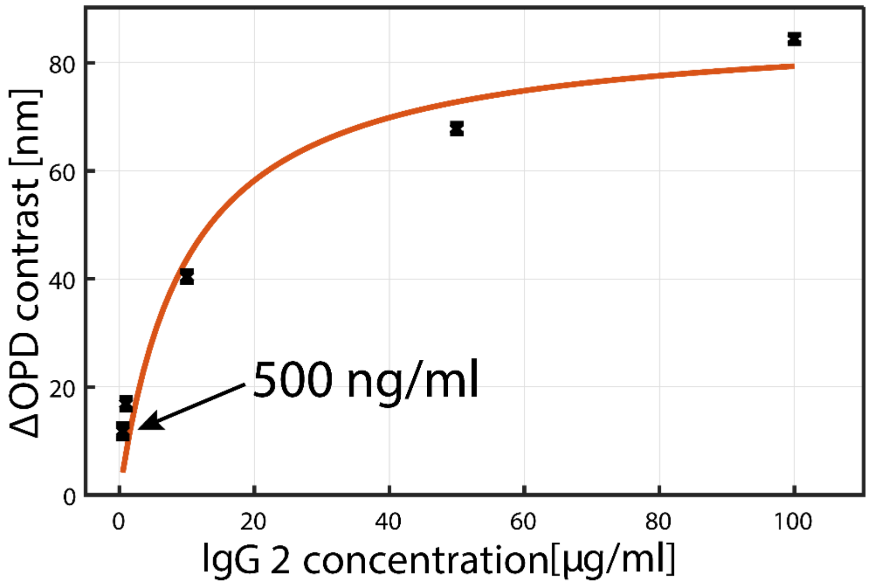

Figure 5S: Calibration curve for IgG 2 measured in dry media. OPD contrast is calculated for each concentration by subtracting the IGG1 contrast from IGG2. Error bars represent the standard deviation where N=36.

Statistical characterization of the fabricated plasmonic chips

In order to understand the effect of the process variations on the relevant plasmonic properties, we evaluated a large set of fabricated Au-NHA plasmonic chips by analyzing their transmission intensity spectra acquired using a conventional spectrophotometer connected to the light outlet of an inverted microscope. When the Au-NHA chips are illuminated by a broadband light source, they transmit a narrow bandwidth corresponding to their EOT resonance, shown in Figure 4S. We extracted the characteristic parameters of the resonance peak, such as its spectral position (λ_{EOT}) and bandwidth, which can be quantified either by considering the full width half max (FWHM) or the half width half max (HWHM) extracted from the sharper side of the curve (see Figure 4S). The results of this statistical study are presented in Table 1S. We measured three spectra per chip, 44 chips per wafer and four wafers, and computed the average standard deviations of the parameters within the chips, wafers and batch. We found that the deviation in the parameters are sufficiently small and only slightly increase from chip to the batch.

Table 1S: Statistically evaluated plasmonic EOT resonance peak properties.

	**Average Value**	**Average STD – per CHIP***	**Average STD – per WAFER***	**STD – ALL***
**λ_{EOT} [nm]**	656.31	0.46	0.95	1.11
**FWHM [nm]**	25.31	1.19	1.32	1.33
**2*HWHM [nm]**	16.73	0.85	1.09	1.11

*3 measurements per chip, 44 chips per wafer, and 4 wafers considered in this study.

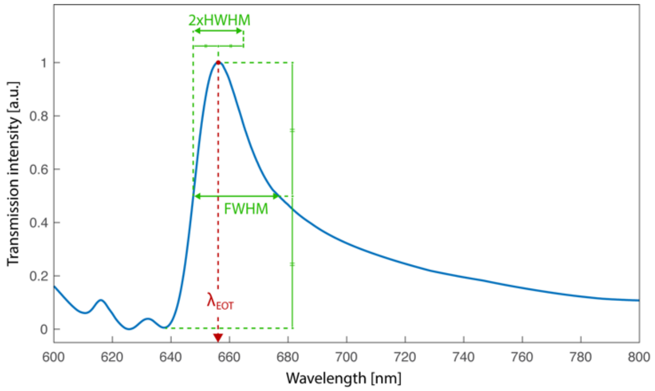

Figure 6S: A typical measured transmission intensity spectra showing the EOT peak. The resonance parameters, such as λ_{EOT} , FWHM and HWHM are indicated on the curve.

References

1. Terborg, R. A., Pello, J., Mannelli, I., Torres, J. P. & Pruneri, V. Ultrasensitive interferometric on-chip microscopy of transparent objects. *Sci. Adv.* **2,** e1600077 (2016).
